# Supplementary figures and images for: Lipid Emulsion Inhibits Amlodipine-Induced Nitric Oxide-Mediated Vasodilation in Isolated Rat Aorta
Source: Int J Mol Sci. 2023 May 14;24(10):8741. doi: 10.3390/ijms24108741 (PMC10218685; doi:10.3390/ijms24108741)

## Slide 1
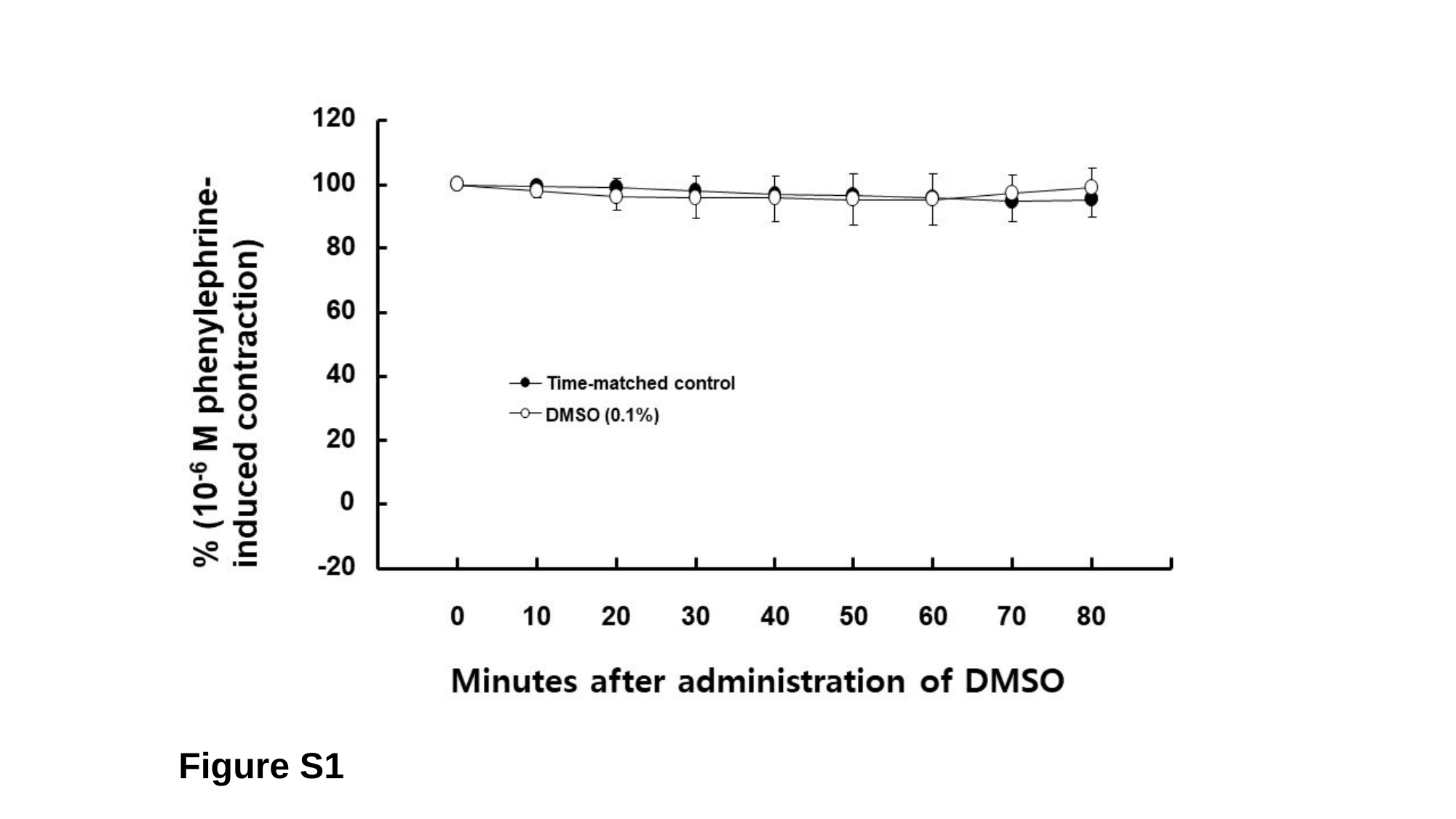

Figure S1

## Slide 2
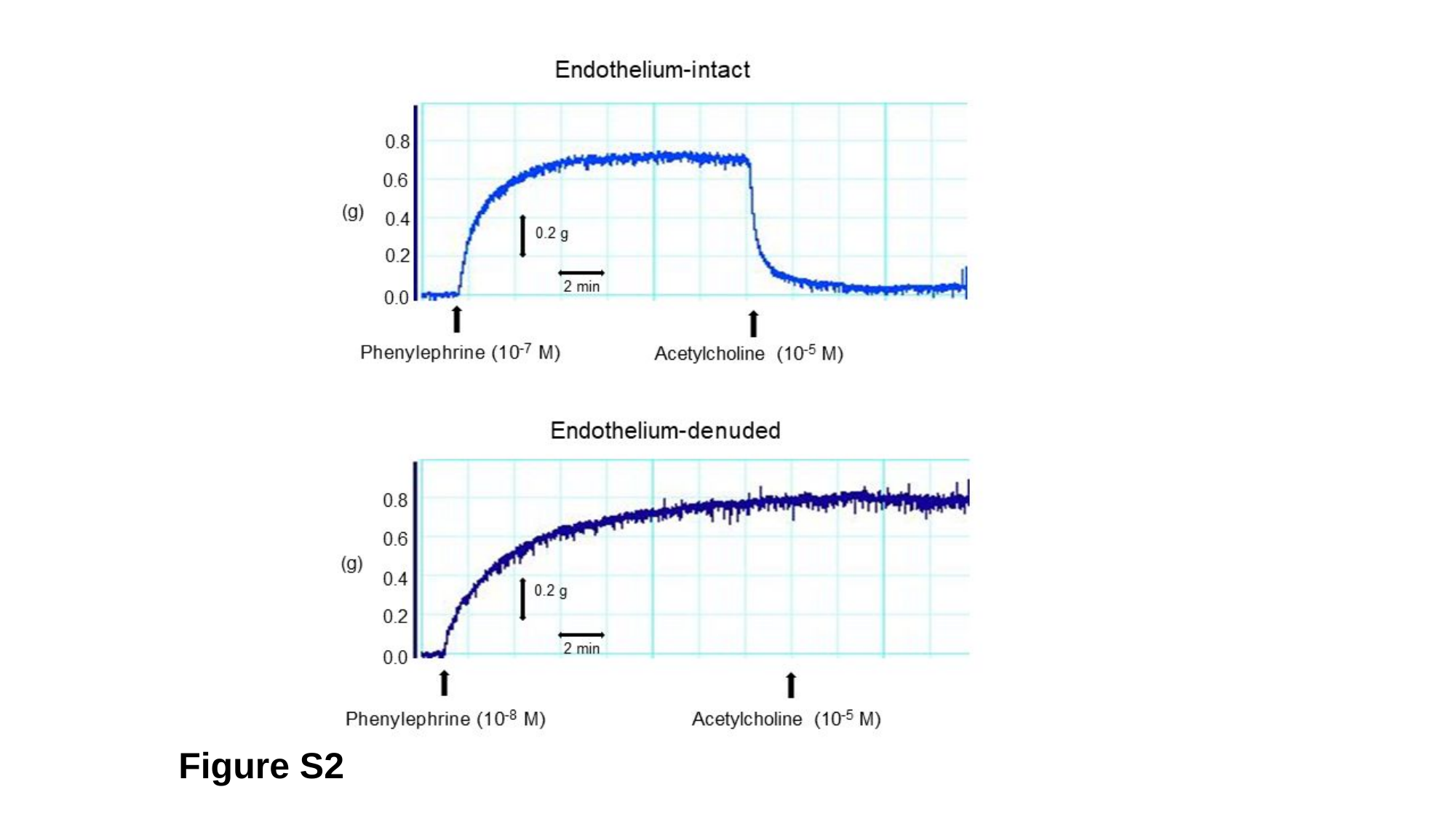

Figure S2

Supplement: Supplementary file 1 [file ijms-24-08741-s001.zip › ijms-2377133-supplementary.pptx]
